# Editorial: Precise Diagnosis and Therapy Using Near-Infrared Light

**DOI:** 10.3389/fbioe.2022.864759

**Published:** 2022-03-01

**Authors:** Yong Fan, Yiqing Lu, Yao Sun

**Affiliations:** ^1^ State Key Laboratory of Molecular Engineering of Polymers, Shanghai Key Laboratory of Molecular Catalysis and Innovative Materials and IChem, Department of Chemistry, Fudan University, Shanghai, China; ^2^ School of Engineering, Faculty of Science and Engineering, Macquarie University, Sydney, NSW, Australia; ^3^ Department of Chemical Biology, Centre Normal University, Wuhan, China

**Keywords:** near-infrared imaging, optical probes, luminescence, biomedical imaging, precise diagnosis

The last decade has witnessed the growing development of optical imaging using near-infrared (NIR) light (wavelength range: 700–1700 nm) in a wide range of applications, from fundamental research of live biological and physiological processes to clinical applications such as disease screening and image-guide surgical interventions. The low-energy NIR light offers reduced tissue scattering, absorption as well as autofluorescence background compared to traditional wavelengths in the visible (400–700 nm) range, thus dramatically enhancing imaging resolution, signal-to-noise ratio, and penetration depth.

This Research Topic calls for advances in new fluorescent probes, imaging instrumentation, analytical methods and their applications for improvement of diagnosis and therapy in this “tissue transparent window,” aiming to provide a timely update of this continuously evolving filed.

Imaging and diagnosis agents are the cornerstone of preclinical applications. In their study, Tan et al. report lycosin-I peptide modified gold nanoclusters (LGNCs), which exhibit strong NIR fluorescence, for imaging of specific cancer cell nuclei. The lycosin-I peptide not only possesses highly efficient and selective intracellular translocation against several cancer cells, but also induces aggregation of gold nanoclusters to further enhance their brightness. After uptake by tumor cells, the LGNCs aggregates can be depolymerized into ultrasmall nanoclusters by the high level GSH in the tumor microenvironment, facilitating nuclear targeting translocation in cancer cells. Another study by Huang et al. reports a novel biomimetic nanoprobe for photoacoustic (PA) imaging of cervical carcinoma. This probe is constructed by coating indocyanine green (ICG)-loaded mesoporous silica nanoparticles with the cancer cell membrane, providing small particle size, excellent dispersion, large loading efficiency, good biocompatibility and homologous targeting ability to Hela cells *in vitro*. The biomimetic nanoprobe can specifically accumulate into tumor and display a superior PA imaging efficacy compared with free ICG, allowing for non-invasive deep tissue imaging of cervical carcinoma *in vivo*.

Another article by Gu et al. focuses on a particular type of NIR fluorophores, semiconducting polymer nanoparticles (SPNs). SPNs are constructed by encapsulating hydrophobic semiconducting polymers (SPs) with amphiphilic copolymers, and have shown great promise for phototheranostics of cancer. The authors review recent advances of thiadiazoloquinoxaline (TQ)-based SPNs from their synthesis and hydrophilic functionalization to NIR-II (1,000–1,700 nm) fluorescence imaging-guided cancer photothermal therapy (PTT). To enhance the PTT efficiency, they also discuss methods for constructing SPNs with capability of multimodal imaging. Current limitations are summarized at the end that hamper clinical applications of the SPNs, such as relatively large size and low tumor targeting ability.

Li et al. reviews the latest novel NIR inorganic nanomaterials and their emission mechanisms, including single-walled carbon nanotubes, rare-earth nanoparticles, quantum dots, and metal nanomaterials. They also discuss key factors of the probes that influence the quality of imaging tissues and organs *in vivo*, such as quantum yield, suitable size, spectral properties and biological compatibility, as well as imaging instruments. The recent progress of precise noninvasive diagnosis in biomedicine and cancer therapy utilizing NIR inorganic nanomaterials are presented. Finally, they envision that multifunctional and stimuli-responsive NIR inorganic nanoparticles as well as new imaging strategies such as bioluminescence or chemiluminescence imaging may need to be pursued to further promote precise diagnosis and therapy.

Cai et al. highlight the recent advances in photothermal, photodynamic and photo-immunotherapy of bone cancer through NIR probes. To provide high-efficiency treatment for bone tumors, NIR probes combined with other customized therapeutic agents are also discussed. Although NIR fluorescence imaging is a highly promising technique for biomedical applications with deeper tissue penetration capability and higher SBR, issues remain to be resolved for clinical diagnosis to improve the targeting ability, to develop probes with immunotherapy function, and to screen new targets for bone tumor.

In another article, Li et al. emphasize recent examples of NIR fluorescence imaging for surgical navigation and the applications in various surgical resection. They first introduce the basic construction and operation principles of fluorescence molecular imaging technology, and then summarize several representative applications, including image-guided surgery of orthotopic osteosarcoma, orthotopic liver tumor, orthotopic breast tumor, renal cell carcinoma, brain tumor, inflammatory bowel disease, peritoneal carcinomatosis, metastatic ovarian cancer, and lymph node. Finally, they discuss present challenges and future perspectives in this field, including how to improve the efficiency of such surgical treatment.

The last article by Zhang et al. summarizes the use of lanthanide-based NIR nanoparticles for super-resolution imaging, benefiting from their unique optical properties of non-photobleaching, sharp emissions and several ladder-like energy levels. In the review, several different mechanisms based on stimulated emission depletion are introduced for various lanthanide ions (Tm^3+^, Er^3+^, Tb^3+^, Eu^3+^, Pr^3+^, and Dy^3+^) to achieve super-resolution imaging. The authors also discuss in detail its applications in imaging nanocrystals themselves, intracellular microtubule structures and cellular cytoskeleton protein desmin. They emphasize the future challenges and outlook, encouraging cooperation with scientists among diverse field, such as chemistry, biology, material science and optics, to accelerate the development of super-resolution imaging in the NIR region.

